# Comparative Analysis of 3D Imaging in Periodontal Disease Assessment: A Systematic Review and Meta‐Analysis

**DOI:** 10.1002/cre2.70169

**Published:** 2025-07-13

**Authors:** Ravinder S. Saini, Sunil Kumar Vaddamanu, Masroor Ahmed Kanji, Seyed Ali Mosaddad, Artak Heboyan

**Affiliations:** ^1^ Department of Allied Dental Health Sciences COAMS, King Khalid University Abha Saudi Arabia; ^2^ Department of Research Analytics, Saveetha Dental College and Hospitals, Saveetha Institute of Medical and Technical Sciences Saveetha University Chennai India; ^3^ Department of Conservative Dentistry and Bucofacial Prosthesis, Faculty of Odontology Complutense University of Madrid Madrid Spain; ^4^ Department of Prosthodontics, School of Dentistry Shiraz University of Medical Sciences Shiraz Iran; ^5^ Department of Prosthodontics, Faculty of Stomatology Yerevan State Medical University after Mkhitar Heratsi Yerevan Armenia

**Keywords:** 3D imaging, CBCT, diagnosis, MRI, periodontology, treatment planning

## Abstract

**Objectives:**

The present systematic review and meta‐analysis aimed to compare the efficacy of three‐dimensional (3D) imaging techniques in terms of accuracy and precision for periodontal disease assessment.

**Material and Methods:**

A literature search was conducted across multiple databases (PubMed, Scopus, Web of Science, Google Scholar, and ScienceDirect) following Preferred Reporting Items for Systematic Reviews and Meta‐analysis (PRISMA) protocols. The primary outcomes focused on comparing the accuracy and precision of 3D versus two‐dimensional (2D) imaging techniques. Furthermore, it assessed their performance in determining periodontal diseases. Quality assessment was performed using the risk of bias (RoB)‐2 for randomized controlled trials (RCTs) and ROB in nonrandomized studies‐Intervention (ROBINS‐I) for non‐RCTs. Meta‐analysis was conducted using RevMan 5.4 with a significance level set at 0.01. While meta‐regression was performed using OpenMEE.

**Results:**

After screening, 22 studies met the eligibility criteria for qualitative and quantitative analysis. Qualitatively, 3D imaging, particularly cone‐beam computed tomography (CBCT), showed superior accuracy and precision over 2D techniques. The meta‐analysis revealed significant differences in several areas: overall (*p* = 0.00001, Mean Difference (MD) = −0.36, 95% confidence interval [CI]: −0.96 to 0.24, *I*² = 93%), horizontal measurements (*p* = 0.00001, MD = −0.75, 95% CI: −2 to −0.49, *I*² = 92%), and vertical measurements (*p* = 0.00001, MD = −0.59, 95% CI: −2.40 to 1.23, *I*² = 92%). Nonsignificant differences were found for furcation height, width, and depth. Most studies showed good quality with a low risk of bias. Age of the participants and study quality were found to be the sources of heterogeneity.

**Conclusions:**

Consistent trends highlight the advantages of 3D imaging in assessing both periodontal and nonperiodontal diseases. However, given the nonsignificant differences in furcation height, width, and depth, the recommended approach is to combine CBCT with digital intraoral radiography for more comprehensive periodontal bone assessment.

## Summary

- 3D imaging is more accurate and precise than 2D imaging or intra‐surgical measurement techniques.
- 3D imaging provides significant advantages in horizontal and vertical disc/plane measurements.
- More research is needed on furcation height, width, and depth, as the results showed no significant difference.

## 1 Introduction

Periodontal diseases are considered inflammatory diseases, and pathogenic bacteria are the causative agents of these diseases (Mosaddad et al. 2019), posing a great challenge to global health sustainability. Conditions related to periodontal diseases range from gingivitis (reversible) to periodontitis (irreversible) and also affect the cementum, alveolar bone, and periodontal tendon, often resulting in tooth loss (Naikwadi and Kale 2024; Xu et al. 2024). Loss of attachment due to periodontal diseases is also correlated with functional limitations (physiological discomfort and physical pain) and may lead to a minimized quality of life (QoL) (Al‐Bitar et al. 2024). Globally, the prevalence of periodontal diseases ranges from 20% to 50% and most commonly occurs in adults and older populations of developing and developed nations (Nazir 2017). Moreover, in the last decade (2011–2020), the prevalence of periodontitis was 62%, with 23.6% of severe and serious forms of periodontitis (Trindade et al. 2023). These diseases increase with increasing age; 70.1% of the population aged > 65 years has some form of periodontal disease (CDC 2024). In addition to periodontal tissue destruction, periodontal diseases also have a significant impact on overall health, as several studies have identified a link that co‐joins periodontal diseases and systematic conditions, including diabetes, rheumatoid arthritis, Alzheimer's disease, inflammatory bowel disease, and pre‐eclampsia (Hajishengallis and Chavakis 2021; Prajjwal et al. 2023). Furthermore, conditions linked to the cardiovascular and pulmonary systems, along with unfavorable consequences during pregnancy, are correlated with periodontal disorders. (Grant et al. 2023). Moreover, different risk factors should be considered before starting any therapy, such as increased age, tooth brushing and flossing (Relvas et al. 2022), smoking (Holmes 2024), alcohol use, ethnic race, and sex (Chang et al. 2024). Meanwhile, in the process of diagnosis, bleeding on probing, periodontal pocket development, gingival tissue recession, furcation involvement, and loss of bone should be assessed (Salvi et al. 2023). Similarly, periapical lesions include apical periodontitis and periapical abscesses, which, if not properly diagnosed, can lead to chronic infections, further bone loss, and systemic complications (van der Waal et al. 2015).

Clinicians have relied on various diagnostic tools to evaluate the extent of tissue damage and monitor disease progression. Among the screening tools, periodontal screening recording is considered a reliable, quick, and reproducible tool for the diagnosis of periodontal disease (Devi et al. 2024). Biomarkers may also play an essential role in the early diagnosis of periodontitis (Blanco‐Pintos et al. 2023; Vaddamanu et al. 2024). For instance, interleukin‐1‐beta and alkaline phosphate are critical salivary biomarkers (Kim et al. 2023). Similarly, the traditional gold standard for assessing periodontal health has been clinical measurements such as probing pocket depth (PPD) and clinical attachment level (CAL) (Ramenzoni et al. 2021). Meanwhile, for better and more comprehensive periodontal evaluation, radiographs serve as a noninvasive method for the assessment of bone loss or damage (Alshadidi et al. 2023; Miller et al. 2023). Different radiographic protocols can be used for evaluation, such as partial mouth recording protocols (de Carvalho‐Buonocore et al. 2023). Two‐dimensional (2D) methods, including panoramic and periapical radiography, are also used for more careful clinical and radiographic assessments of periodontal disease (Asmita et al. 2014). 2D radiographs, while widely used, offer limited information regarding the three‐dimensional (3D) anatomy of the periodontium (Komšić et al. 2019). Radiographs provide a flat image of complex structures, making it difficult to assess bone defects accurately, especially in the initial parts of periodontal disease (Shah 2014). Likewise, the detection of periapical lesions can be hindered by the superimposition of anatomical structures in 2D radiographs, particularly in posterior teeth where surrounding bone or roots can obscure the lesion (Lo Giudice et al. 2018). Other challenges, such as 2D projection, distortion, magnification, misrepresentation of anatomical structures, and superimposition, discourage the use of 2D radiography (Chakrapani et al. 2013). For better correlation between radiographic evaluation and clinical findings, 3D) imaging is more helpful for accurate and precise diagnosis (Hassan et al. 2023). Examples include cone beam computed tomography (CBCT) (Gupta et al. 2015; Leonardi Dutra et al. 2016) and magnetic resonance imaging (MRI) (Johannsen et al. 2023). Furthermore, periodontal ligament 3D digital models developed by CBCT provide accurate and precise information regarding the anatomy of the alveolar crest and facilitate reproducible measurements (Lyu et al. 2023). When CBCT radiographs of segmented periapical lesions (PALs) of apical periodontitis were validated through 3D PAL‐Net (a neural network algorithm), they showed more robustness, improved the diagnostic performance of dentists, and provided more detailed information (Fu et al. 2024). In addition to accuracy, 3D radiographs, particularly CBCT, are considered low‐cost and require low doses compared to medical fan‐beam CT (Mohan et al. 2011). Moreover, MRI generates a high contrast for tissues and provides more detailed soft‐tissue images. It also provides more information on processes related to the inflammation of hard and soft tissues (Johannsen et al. 2023; Tymofiyeva et al. 2009).

Therefore, the aim of this systematic review and meta‐analysis was to compare the efficacy of 3D imaging techniques (e.g., CBCT and MRI) in terms of accuracy and precision for periodontal disease assessment, including periapical lesions. Through this comparison, the study identified the strengths, limitations, and challenges associated with 3D and 2D radiography for the evaluation of periodontal diseases. Furthermore, the potential clinical implications of each method will aid dentists in making better decisions regarding the optimal diagnosis of periodontal disease and periapical lesions.

## 2 Materials and Methods

This study project complied with the Preferred Reporting Items for Systematic Reviews and Meta‐Analyses (PRISMA) guidelines to ensure integrity and consistency throughout the literature exploration. (Arya et al. 2021). The protocol used for this systematic review was registered at the International Platform of Registered Systematic Review and Meta‐Analysis Protocols (INPLASY202420063).

### 2.1 Search Strategy

The main goal of the research was to compare the effectiveness of 3D techniques for assessing periodontal disease, particularly diagnostic accuracy and reliability. The structure provided by PICO facilitated the selection of the subsequent research articles: Population (P): Adults diagnosed with periodontal disease; Intervention (I): Utilization of a 3D approach for evaluation; Comparison (C): Any alternative 2D‐methodologies; Outcomes (O): Accuracy, accuracy, and dependability of 3D imaging methodologies. A comprehensive search of databases, including PubMed, ScienceDirect, Scopus, Web of Science, and Google Scholar, was conducted from January 2003 to April 2024, and the same search terms were used in all the databases, utilizing predetermined keywords: “three‐dimensional imaging” OR “3D imaging” OR “cone beam computed tomography” OR “CBCT” OR “magnetic resonance imaging” OR “MRI” OR “digital volumetric tomography” AND “periodontal disease” OR “periodontitis” OR “periapical disease” OR “furcation defects” OR “periapical lesion” OR “apical periodontitis” OR “alveolar bone loss” OR “intrabony defects” (Table S1).

### 2.2 Eligibility Criteria

The inclusion and exclusion criteria were based on PICO research questions. Nonetheless, distinct inclusion criteria were established for the papers incorporated in the current research: studies performed on live humans aged > 18 years focused on 3D imaging such as CBCT and MRI used for the assessment of periodontal and nonperiodontal (e.g., apical periodontitis, periapical abscesses) diseases in comparison with 2D or any other technique as a control; studies report diagnostic accuracy, precision, or clinical outcomes associated with 3D imaging in assessing periodontal and nonperiodontal diseases, followed by randomized controlled trials (RCTs), non‐RCTs, published in the English language.

Exclusion criteria were applied, eliminating studies conducted on animals, cadaveric human skulls, teeth, or patients under 18 years old, as well as those lacking comparison or control groups. Additionally, studies utilizing artificial intelligence‐based imaging methods for assessment were excluded. Studies that focused on various oral health concerns unrelated to periodontal or periapical disease, such as dental caries or orthodontic issues, without a relevant diagnostic comparison. Research focussing on bone conditions for dental implants and investigations emphasizing the application of CBCT in the assessment of artificially induced bone deficiencies reviews (systematic, narrative, scoping, literature), expert opinion, letters, conference abstracts, editorial, commentary, or case studies.

### 2.3 Study Selection, Assessment, and Data Extraction

Two independent reviewers selected the research papers. First, they screened the titles and abstracts of each research paper in accordance with our aim. After the initial screening, full‐text screening was performed, and research papers were selected after meeting the inclusion criteria. A third reviewer was also engaged in case of any disagreement, and concerns were addressed through thorough discussions and assessment of the eligibility criteria.

The data extraction emphasized specified parameters: study features (study ID, country, World Health Organization [WHO] region, design of the study, size of sample, total number of teeth), characteristics of patients (sex ratio, age, teeth, disease type, prevalence), details of 3D radiography examination (3D imaging technique, software utilized for 3D, resolution/scan parameters, image assessment, image display, review), and outcomes (diagnostic evaluation, major outcomes, conclusions, and restrictions).

### 2.4 Quality Assessment

RCT quality was assessed using the Cochrane Collaboration's Robvis web application, focusing on five domains: randomization process, deviations from intended interventions, data measurement, missing outcomes, and reporting. For nonrandomized controlled trials, the Risk of Bias (RoB) assessment utilized the ROB In nonrandomized Studies‐Intervention (ROBINS‐I) framework, which evaluates seven domains: confounding, participant choice, intervention classification, bias, incomplete outcome data, measurement of outcomes, and outcome reporting. Two authors performed the quality assessment of the studies, and in case of controversy, a third senior author was consulted to resolve the issue.

### 2.5 Statistical Analysis

The extracted data were transferred to an Excel sheet for organization and construction of tables and graphs. For inter‐reviewers agreement, Cohen's kappa statistic was performed using SPSS‐16. While RevMan 5.4 was utilized for the meta‐analysis, and the mean and standard deviation of the accuracy or assessment tool of 3D and control (2D, intra‐surgical interventions for measurement) radiographs for the assessment of periodontal disease were used. A confidence interval of 95% was used to measure the effect size. The Cochrane Q test and *I* ^*2*^ were used to analyze the heterogeneity among the studies. Heterogeneity was assessed as low, medium, or high when *I* ^*2*^ was < 25%, 25%–50%, or > 50%, respectively. Meanwhile, meta‐regression for the source of heterogeneity was performed using OpenMEE. The chi‐square test was employed to evaluate the difference, deemed significant at *p* < 0.01. A funnel plot was also constructed for publication bias.

## 3 Results

### 3.1 Literature Search

**Figure 1:**
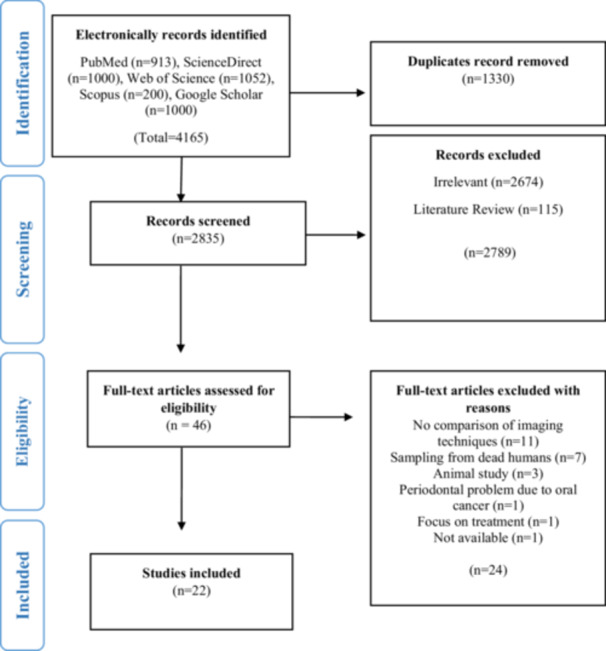
PRISMA flow chart and article selection process.

The literature search conducted across PubMed, ScienceDirect, Web of Science, Scopus, and Google Scholar initially identified 4165 papers. During the first PRISMA phase (identification), 1330 duplicate articles were identified. In the second step (title and abstract screening), 2835 papers were examined for eligibility for the systematic review and meta‐analysis. Following a comprehensive review, 2789 research papers were eliminated. Approximately 46 research papers advanced to the full‐text evaluation stage (third phase of PRISMA). In total, 22 papers were incorporated for qualitative and quantitative analysis, but 24 were excluded for various reasons (Figure 1). The whole process was performed by two independent reviewers, and the inter‐reviewer agreement was significantly very high, with Cohen's kappa value of 0.97.

### 3.2 Distribution of Research Papers Over Countries and WHO Regions

**Figure 2:**
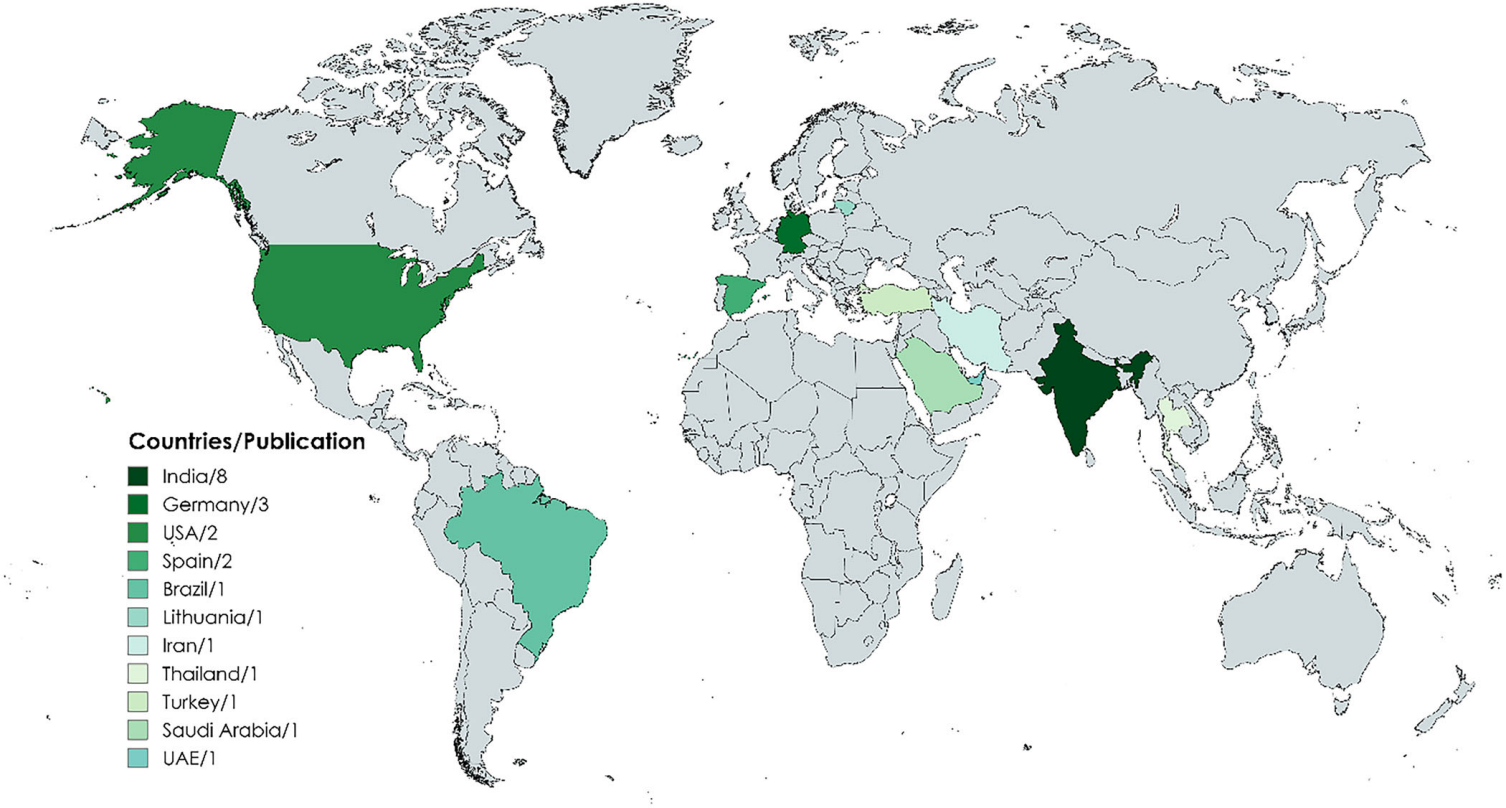
Worldwide distribution of selected studies.

Most of the studies were reported in India (Adurty et al. 2021; Das and Adhikari 2021; Mark et al. 2021; Padmanabhan et al. 2017; Patil et al. 2023; Patil et al. 2018; Pitale et al. 2020; Sheth et al. 2020), which is in the Southeast Asian Region (SEAR) of the World Health Organization (WHO), followed by Germany (Feuerriegel et al. 2023; Schloss et al. 2017; Yusof et al. 2021) in the European Region (EUR), the United States of America (USA) (Balasundaram et al. 2012; Zhang et al. 2018), Brazil (Estrela et al. 2008) in the Region of Americas (AMR), Spain (Abella et al. 2013; Ramis‐Alario et al. 2019), Lithuania (Venskutonis et al. 2014), Turkey (Keser and Namdar Pekiner 2018) in EUR, Thailand (Suphanantachat et al. 2017) in SEAR, Iran (Moradi Haghgoo et al. 2014), Saudi Arabia (Alsaikhan et al. 2022), and the United Arab Emirates (UAE) (Alshamsi et al. 2023) in the Eastern Mediterranean Region (EMR), as shown in Figure 2 and Table 1.

**Figure 3:**
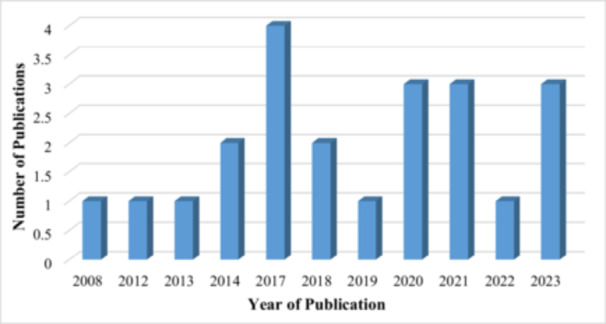
Number of studies over time.

Moreover, most of the studies were published in 2017 (4), and an equal number of studies (3) were reported in 2020, 2021, and 2023, as indicated in Figure 3.

**Table 1:** Summary of general characteristics of included studies.

Demographical characteristics						Patient characteristics				
Study ID	Country	WHO region	Study design	Sample size	No. of teeth	Gender (M:F)	Age	Teeth	Disease type	Prevalence
**Nonperiodontal disease**										
Estrela et al. (2008)	Brazil	AMR	Comparative (non‐RCT)	888	1508	364:524	50	Premolar, Molars, Canines, Incisors	Apical periodontitis	For treated: CBCT = 63.3%, panoramic = 17.6%, periapical = 35.3%; For untreated CBCT = 74.7%, panoramic = 21.7%, periapical = 36.1%
Balasundaram et al. (2012)	USA	AMR	Comparative (non‐RCT)	24	24	13:11	53	Maxillary/mandibular tooth	Periapical lesions	NA
Abella et al. (2013)	Spain	EUR	Comparative (non‐RCT)	155	161 (340 roots)	71:84	47	Maxillary/mandibular tooth	Periapical radiolucencies	Apical periodontitis = 55, Symptomatic apical periodontitis = 50, Chronic apical abscess = 25, Asymptomatic acute apical abscess = 16, Pulp necrosis with normal apical tissue = 15
Venskutonis et al. (2014)	Lithuania	EUR	Comparative (non‐RCT)	20	35	13:7	42.4	Maxillary/mandibular tooth	Periapical lesion	100%
Schloss et al. (2017)	Germany	EUR	Comparative (non‐RCT)	44	51	18:26	45.9	Maxillary/mandibular tooth	Periapical lesion	100%
Keser and Namdar Pekiner (2018)	Turkey	EUR	Comparative (non‐RCT)	200	NA	100:100	37.9	Root‐filled teeth	Periapical lesion	27%
Ramis‐Alario et al. (2019)	Spain	EUR	Comparative (non‐RCT)	35	45	18:17	47	Premolar, MolarLower and upper incisors, Canines	Periapical disease	100%
Sheth et al. (2020)	India	SEAR	Comparative (non‐RCT)	20	NA	14:6	23.9	Single‐rooted maxillary anterior teeth	Periapical lesions	100%
Das and Adhikari (2021)	India	SEAR	Comparative (non‐RCT)	35	NA	23:12	26.5	Anterior teeth	Periapical lesions	100%
Alsaikhan et al. (2022)	Saudi Arabia	EMR	Comparative (non‐RCT)	72	204	43:29	NA	NA	Periapical lesions	CBCT = 95.10%; Pa = 63.73%
Feuerriegel et al. (2023)	Germany	EUR	Comparative (non‐RCT)	37	232	19:18	62	Viscerocranium	Apical periodontitisReactive bone edema	75%
**Periodontal diseases**										
Moradi Haghgoo et al. (2014)	Iran	EMR	Comparative (non‐RCT)	50	NA	NA	NA	NA	Periodontal osseous lesions	100%
Suphanantachat et al. (2017)	Thailand	SEAR	Comparative (non‐RCT)	25	666	11:14	48.8	MolarPremolarCaninesIncisors	PeriodontitisInfrabony defects	79%
Padmanabhan et al. (2017)	India	SEAR	Comparative (non‐RCT)	14	NA	NA	20‐60	Mandibular Molar	Furcation Involvement	100%
Zhang et al. (2018)	USA	AMR	Comparative (non‐RCT)	80	NA	28:52	54.9	CaninesCentral incisorsFirst molars of the left mandible and right maxilla	Periodontal diseases	NA
Patil et al. (2018)	India	SEAR	Comparative (non‐RCT)	32	NA	18:14	40	PremolarMolar	Intrabony defects	100%
Pitale et al. (2020)	India	SEAR	Comparative (non‐RCT)	12	144	NA	NA	Anterior and posterior	Chronic periodontitis	100%
Yusof et al. (2021)	Germany	EUR	RCT	22	NA	13:9	44‐47.8	Maxillary and mandibular molar	Furcation defects	100%
Adurty et al. (2021)	India	SEAR	Comparative (non‐RCT)	25	NA	NA	NA	NA	Periodontitis Intrabony defects	100%
Mark et al. (2021)	India	SEAR	Comparative (non‐RCT)	10	NA	NA	NA	NA	Periodontal defects Furcation involvement	100%
Alshamsi et al. (2023)	UAE	EMR	Comparative (non‐RCT)	22	135	NA	30‐65	Molar maxillary and mandibular	Periodontitis (furcation involvement)	NA
Patil et al. (2023)	India	SEAR	Comparative (non‐RCT)	40	NA	NA	46.2	Anterior and posterior	Chronic periodontitis	100%

Abbreviations: AMR, region of the Americas; CBCT, cone beam computed tomography; EUR, European Region; EMR, Eastern Mediterranean Region; F, female; M, male; NA, not available; Pa, periapical radiograph; RCT, randomized controlled trial; SEAR, South‐East Asian Region.

### 3.3 General Characteristics and Summary of Included Studies

#### 3.3.1 Nonperiodontal Diseases

All studies followed a non‐RCT study design. There were 1530 patients included in the selected studies, with a minimum number of 20 (Sheth et al. 2020; Venskutonis et al. 2014), while 888 were included in the maximum number of patients included by Estrela et al. (2008). Overall, 2260 teeth were studied to assess the efficacy of different diagnostic interventions, with a minimum number of teeth of 24 (Balasundaram et al. 2012) and a maximum number of 1508 (Estrela et al. 2008). The majority of the studies included both genders for better comparison and response, with the minimum number of males (13) included by Balasundaram et al. (2012), while the minimum number of females was 5 (Venskutonis et al. 2014). Similarly, the maximum numbers of males and females were 364 and 524, respectively (Estrela et al. 2008). Diagnostic interventions were evaluated using almost all types of teeth, including maxillary and mandibular teeth, premolars, molars, and incisors (Table 1). The majority of the studies included 100% of patients with any type of nonperiodontal disease; however, periapical lesions were the most prevalent nonperiodontal condition reported in most studies (Alsaikhan et al. 2022; Balasundaram et al. 2012; Das and Adhikari 2021; Keser and Namdar Pekiner 2018; Schloss et al. 2017; Sheth et al. 2020; Venskutonis et al. 2014). Meanwhile, some of the studies also reported confirmed patients with the use of diagnostic interventions such as CBCT and other comparative interventions (2D radiographs) (Table 1).

#### 3.3.2 Periodontal Diseases

All studies followed a non‐RCT study design, except one study that followed an RCT design (Yusof et al. 2021). There were 332 patients included in the selected studies, with a minimum number of 10 (Mark et al. 2021), while 80 were included in the maximum number of patients included by Zhang et al. (Zhang et al. 2018). Overall, 945 teeth were studied to assess the efficacy of different diagnostic interventions, with a minimum number of teeth of 135 (Alshamsi et al. 2023) and a maximum number of 666 (Suphanantachat et al. 2017). The majority of the studies included both genders for better comparison and response, with the minimum number of males (11) included by Suphanantachat et al. (2017), while the minimum number of females was 9 (Yusof et al. 2021). Similarly, the maximum numbers of males and females were 28 and 52, respectively (Zhang et al. 2018). Diagnostic interventions were evaluated using almost all types of teeth, including premolars, molars, incisors, maxillary, and mandibular teeth (Table 1). The majority of the studies included 100% of patients with any type of periodontal disease (Table 1).

### 3.4 Intervention Characteristics

#### 3.4.1 Nonperiodontal Diseases

In the study, CBCT emerged as the frequently used 3D diagnostic tool for assessing periodontal diseases, with the exception of one study that used MRI (Feuerriegel et al. 2023). Differences were noted in the program utilized for analyzing the images generated by these 3D diagnostic instruments; however, i‐CAT viewing software was predominantly adopted. (Balasundaram et al. 2012; Sheth et al. 2020; Venskutonis et al. 2014). The images from 3D (CBCT and MRI) were evaluated by professionals and trained people, as shown in Table 2, with all the studies involving at least two examiners. Images were analyzed for various periodontal conditions, with most studies measuring axial, coronal, and sagittal discs/planes to aid in the treatment (Abella et al. 2013; Venskutonis et al. 2014; Zhang et al. 2018). Additionally, panoramic and periapical radiography were commonly used for comparison, and intra‐operative ultrasound was also utilized in some studies (Yusof et al. 2021; Zhang et al. 2018) (Table 2).

**Table 2:** Summary of intervention and control characteristics.

Study ID	3D radiographic examination characteristics					
	3D intervention	Software/System used	Resolution/scanning parameters	Image assessment	Images view	Control
**Nonperiodontal disease**						
Estrela et al. (2008)	CBCT	3D tomo X (version 1.0.51)	Voxel size = 0.125 mm, 12 or 8 bits	Three examiners	Periapical structures, changes in bone structure, radiolucent area, mineral loss, exacerbating features	Periapical and panoramic radiographs
Balasundaram et al. (2012)	CBCT	i‐CAT (Imaging Sciences International, Hatfield, PA, USA)	Isotropic voxel size = 0.25 × 0.25 × 0.25 mm, 14 bits; Pulsed exposure = 120 kVp, 3 to 7 mA; Exposure time = 14.7 s	Six endodontists worked as observers	Only the arch	Intraoral periapical radiograph
Abella et al. (2013)	CBCT	ProMax 3Ds (Planmeca OY, Helsinki, Finland)	Pulsed exposure = 8.0 mA and 84 kV; Exposure time = 12 s; FOV = 598 cm	Two calibrated endodontists	Axial, coronal, and sagittal	Periapical radiographs
Venskutonis et al. (2014)	CBCT	i‐CAT (Imaging Sciences International Inc., Hatfield, PA, USA)	Pulsed exposure = 120 kVp, 3–8 mA; voxel resolution = 0.2 mm; field view = 6 × 16 cm; acquisition time = 26.9 s	Two endodontists	Sagittal, coronal, and axial planes	Digital periapical radiography
Schloss et al. (2017)	CBCT	i‐Dixel 2.0 (J Morita Mfg Corp)	Pulsed exposure = 80 kV, 5.0–8.0 mA; a voxel size = 0.125 mm; Exposure time = 9.4 s	Two faculty members	Volume changes of periapical lesions	2‐dimensional (2D) periapical films
Keser and Namdar Pekiner (2018)	CBCT	NA	NA	Radiologist	Axial, coronal, and sagittal axis	Panoramic radiographs
Ramis‐Alario et al. (2019)	CBCT	Planmeca Romexis Viewer	Pulsed exposure = 90 kV, 10 mA; Voxel size = 0.15 mm; FOV = 40 × 40 mm	Two blinded examiners	The distance between the mandibular canal's upper border and the first molar's mesial root apex.	Periapical and panoramic radiographs
Sheth et al. (2020)	CBCT	i‐CAT	Pulsed exposure = 120 kVp, 5 mA; Exposure time = 7 s; FOV = 8 × 5 cm	Three endodontists	Anatomical structures	Periapical radiographs
Das and Adhikari (2021)	CBCT	SkyView (My‐RayDental Imaging, Imola, Italy)	Pulsed exposure = 90 kV, 10 mA with gray levels of 4096 (12 bit)	Two blinded observers	Both arches	Intra‐oral periapical radiography and ultrasound
Alsaikhan et al. (2022)	CBCT	Galileo system	NA	Investigators	Axial, coronal, and sagittal axis	Periapical radiographs
Feuerriegel et al. (2023)	MRI	A 3D short‐tau‐inversion‐recovery sequence	Voxel size (acquisition), 0.65 × 0.65 × 1.0 mm^3^; Acquisition time = 6.03 min	A dentist and radiologist	Axial, coronal, and sagittal axis	Panoramic radiography and periapical radiographs
**Periodontal diseases**						
Moradi Haghgoo et al. (2014)	CBCT	NNT	Pulsed exposure = 110 kVp, 10.65 mAs; FOV = 6; pixel size = 200 μm	Two investigators	Coronal and sagittal plans	Digital Direct Intraoral Radiography
Suphanantachat et al. (2017)	CBCT	One Volume Viewer	Cylindrical volumes = 100 × 100 mm; Pulsed exposure = 80 kV, 5 mA; voxel size of 0.25 mm; exposure time = 17.5 s	Three periodontists	Full‐mouth periapical radiographs and vertical bitewings of the posterior teeth	Conventional Intraoral Radiography
Padmanabhan et al. (2017)	CBCT	Kodak (Oblique view, Care stream 3D Imaging Software (Version 3.1)	Pulsed exposure = 84 kV, 5 mA; Exposure time=20 s; Voxel size=180 μm	Two calibrated examiners	Axial, sagittal, and coronal	Direct Surgical Measurements
Zhang et al. (2018)	CBCT	Anatomage Invivo	FOV = 150 × 9 × 90 mm^2^; Pulsed exposure = 90 kVp, 10 mA; Exposure time = 16 s; Voxel size = 0.2‐mm^3^	Different examiners	Cross‐sectional and sagittal views	Clinical and intraoral radiographic examinations
Patil et al. (2018)	CBCT	NA	NA	NA	Mesiodistal width of the periodontal defect	Periapical radiographs and intra‐surgical measurements
Pitale et al. (2020)	CBCT	NNT (Version 5.4, CEFLA Dental Group, Newtom GiANO, Italy)	Pulsed exposure = 90 kV, 10 mA; voxel size = 400 μm	NA	Buccal, Palatal/lingual, Mesial, and distal sites,	Surgical measurements
Yusof et al. (2021)	CBCT	CS 9000 3D proprietary	Pulsed exposure = 78 kV, 10 mA; Voxel size = 0.076 × 0.076 × 0.076 mm; FOV = 5 × 3 cm	Four trained, calibrated examiners	Root trunk (RT), BL‐H (depth), BL‐V (height), CEJ‐BD (clinical attachment loss), and FW (width))	Periapical radiographs and intra‐surgical linear measurement techniques
Adurty et al. (2021)	CBCT	Image processing software from Care Stream Health (CS 9300, version 2.2, New York, USA)	Voxel size = 90 μm; Acquisition time = 12 s; FOV = 5 × 5 cm	A radiology clinician and specialist	Height, depth, width, and angle	Digital intraoral periapical radiography
Mark et al. (2021)	CBCT	DICOM	Pulsed exposure = 80 kVp, 4 mAs	Blinded trained periodontist	Mesial and distal sites of the buccal aspect of the defects	Direct digital radiography
Alshamsi et al. (2023)	CBCT	ProMax 3D Mid device (Planmeca)	Pulsed exposure = 90 kVp, 8 mA; Exposure time = 12 s; Voxel size = 200 mm; FOV = 100 × 85 mm	One examiner	Both arches	Curved Nabers probe
Patil et al. (2023)	CBCT	In vivo software (Anatomage, San Jose, USA)	Pulsed exposure = 90 kV, 10 mA; Isotropic voxel size = 0.125 mm^3^; Exposure time = 13 s	Periodontist (15 years of experience) and a trained radiologist (18 years of experience)	Buccal/labial, lingual/palatal, mesial and distal aspects of the defects	Direct surgical measurement

Abbreviations: CBCT, cone beam computed tomography; CS, Care Stream; DICOM, digital imaging and communication in medicine; FOV, field of view; kVp, kilovoltage peak; mAs, milliampere‐s; s, second; MRI, magnetic resonance imaging.

#### 3.4.2 Periodontal Diseases

Similarly, CBCT was singled out as the primary 3D diagnostic tool for periodontal disease assessment (Table 2). There were differences in the software used to view the images, with NNT being the most frequently used [43,59]. The images from CBCT were assessed by trained professionals, as highlighted in Table 2 (Alshamsi et al. 2023). Images were evaluated for various periodontal conditions, with most studies measuring axial, coronal, and sagittal planes for treatment (Table 2). Panoramic and periapical radiography were commonly used for comparison, and surgical techniques were also employed in some studies (Alshamsi et al. 2023; Patil et al. 2023, 2018; Pitale et al. 2020) (Table 2).

### 3.5 Outcomes

#### 3.5.1 Nonperiodontal Diseases

Diagnostic assessment scoring methodologies in the included research were inadequately supported. Many approaches have been identified, including the Periapical Index (PAI) grading system [50, 53, 57] and White and Pharoah's approach [46]. Overall, the studies observed that 3D imaging is more accurate than 2D and intrasurgical procedures for the diagnosis of nonperiodontal disease (Table 3). However, the study also reported no differences between the modalities tested [51]. Additionally, another study found that ultrasound gave higher accuracy than CBCT and sensitivity [46]. The shortcomings of every study are summarized in Table 3.

#### 3.5.2 Periodontal Diseases

The diagnostic assessment score system employed in the included research lacked substantial evidence. However, systems such as CAL [49, 52, 58] and the Glickman classification [61] have been identified. Overall, the studies observed that 3D imaging is more accurate than 2D and intrasurgical procedures for the diagnosis of periodontal disease (Table 3). Some studies have shown similar findings between the modalities tested [41, 44]. The limitations of each study are summarized in Table 3.

**Table 3:** Summary of outcomes.

Study ID	Outcomes (Accuracy, precision, reliability)			
	Diagnostic assessment	Key findings	Conclusion	Limitations
**Nonperiodontal disease**				
Estrela et al. (2008)	PAI	According to PAI scores, CBCT provides a better diagnosis of AP than conventional images.	CBCT was proved to be accurate	NA
Balasundaram et al. (2012)	NA	A nonsignificant (*p* > 0.05) difference was observed among the two diagnostic techniques (χ^2^ = 0.036).	CBCT and 2D radiographs had similar outcomes	Small sample size, absence of patient's medical history, experience level of observers varied, radiation dose, resolution
Abella et al. (2013)	NA	CBCT revealed 67 (19.7%) roots with PA radiolucencies, whereas PA radiographs did not, indicating a significant difference (*p* < 0.05) between the two methods.	CBCT was more accurate	NA
Venskutonis et al. (2014)	NA	In terms of periapical radiolucencies, a statistically significant disparity (*p* < 0.05) was noted between the radiographic (*n* = 24) and CBCT (*n* = 42) checks.	CBCT was more accurate	NA
Schloss et al. (2017)	NA	The 2D and 3D healing classifications were significantly different (*p* < 0.05)	Imaging with CBCT allowed a more precise evaluation	NA
Keser and Namdar Pekiner (2018)	PAI	A significant difference (*p* < 0.01) and positive correlation was found between the two modalities.	CBCT provides more accurate information	NA
Ramis‐Alario et al. (2019)	NA	The periapical and panoramic radiography (2D techniques) yielded a 2% sensitivity, while in the case of CBCT, it was 100%	CBCT was more accurate	Variability of vertical and horizontal magnification
Sheth et al. (2020)	NA	A notable disparity (*p* < 0.05) was noted in the treatment strategy utilizing the two approaches.	CBCT provides better accuracy	NA
Das and Adhikari (2021)	Based on the White and Pharoah approach	IOPA radiographs had accuracy of 62.8% and 54.29%, CBCT had 68.57% and 71.43%, and ultrasound had 88.57% and 94.28% for the diagnosis of cystic lesions and granuloma, respectively.	Ultrasound was found to be a more accurate tool	NA
Alsaikhan et al. (2022)	NA	To diagnose periapical lesions, there is a notable disparity (*p* < 0.05) between CBCT radiographs and periapical radiographs.	CBCT is better than periapical radiographs	Small sample size, resolution
Feuerriegel et al. (2023)	PAI	Significantly (*p* = 0.02) a higher PAI score on MRI (STIR 1.9 ± 0.7) than OPT (1.2 ± 0.7) and dental radiographs (1.3 ± 0.5)	MRI was more feasible	Small sample size and heterogenous, prolonged exposure time
**Periodontal diseases**				
Moradi Haghgoo et al. (2014)	NA	A significant difference (*p* = 0.0001) between the discrepancy from the mean of the gold standard (0.53 ± 0.59) was lower in CBCT (0.56 ± 0.45)	Accuracy was found higher with CBCT in evaluating periodontal bony defect's vertical dimensions	NA
Suphanantachat et al. (2017)	CAL and radiographic bone loss	There was a greater rate of full agreement for CBCT than IOR across the board for all evaluations, and the inter‐examiner agreement was excellent (Fleiss' kappa 0.87–0.94).	CBCT was superior and accurate to IOR	NA
Padmanabhan et al. (2017)	NA	A nonsignificant (*p* > 0.05) difference was observed for CBCT and direct surgical measurements.	CBCT was comparable in terms of accuracy to that of direct surgical measurements	NA
Zhang et al. (2018)	CAL	The correlations between CBCT, PA/BW, and CAL measures were positive and statistically significant (*p* = 0.001).	CBCT is validated for periodontal assessment	Different examiners were used for the CAL
Patil et al. (2018)	NA	The measures of the faciolingual width and MD width of the defect, taken by CBCT and surgical methods, did not differ significantly (*p* > 0.05). However, there was a notable disparity (*p* < 0.05) in the measures taken from the CEJ to the BD and the distance from the bone crest to the deepest point of the defect.	CBCT and intra‐surgical measurements are similar	NA
Pitale et al. (2020)	NA	The mean CBCT and surgical value of the distal locations of anterior teeth and palatal/lingual teeth were significantly different (*p* = 0.001). Regarding the back teeth, however, there was no notable change (*p* < 0.05).	Regarding OFD, both CBCT and clinical assessment demonstrated comparable accuracy in assessing the bone topography. CBCT offers excellent visibility for visualizing areas such as posterior teeth, palatal sites, and distal sites.	NA
Yusof et al. (2021)	CAL	No statistically significant differences (*p* > 0.05) were identified between CBCT and intra‐surgical linear measures for any clinical variables. An evident disparity (*p* < 0.05) was noted in BL‐V readings. CBCT had a sensitivity of 62.8%, whereas periapical radiography demonstrated a sensitivity of 56.9%.	CBCT provided better diagnostic and sensitivity	An investigation was carried out on the deepest area of a molar exhibiting furcation abnormalities.
Adurty et al. (2021)	NA	The average intergroup comparison values between digital IOPA and CBCT exhibited dissimilarities.	Digital IOPA with digital software can be used as an alternative to CBCT	Small sample size, short study period
Mark et al. (2021)	NA	No statistically significant difference (*p* > 0.05) was observed between the tested modalities.	CBCT provides information more accurately and precisely	NA
Alshamsi et al. (2023)	Glickman's classification for FI	CBCT detected grade III FI compared to clinical probing	CBCT was more reliable	The classification system used, the higher the dose of radiation
Patil et al. (2023)	NA	Between the two techniques, a statistically significant disparity (*p* < 0.05) was noted.	CBCT provides an accessibility advantage by enhancing visual access to challenging sites	NA

Abbreviations: 2D, two‐dimensional;‐dimensional; 3D, three‐dimensional; BW, bitewing; CAL, clinical attachment loss; CBCT, cone beam computed tomography; CEJ, cementoenamel junction; IOPA, intraoral periapical radiography; IOR, intra‐oral radiography; MRI, magnetic resonance imaging; OFD, open flap debridement; OPT, conventional panoramic radiography; PAI, periapical index; PA, periapical; STIR, short tau inversion recovery.

### 3.6 Meta‐Analysis (Sub‐Group Analysis)

**Figure 4:**
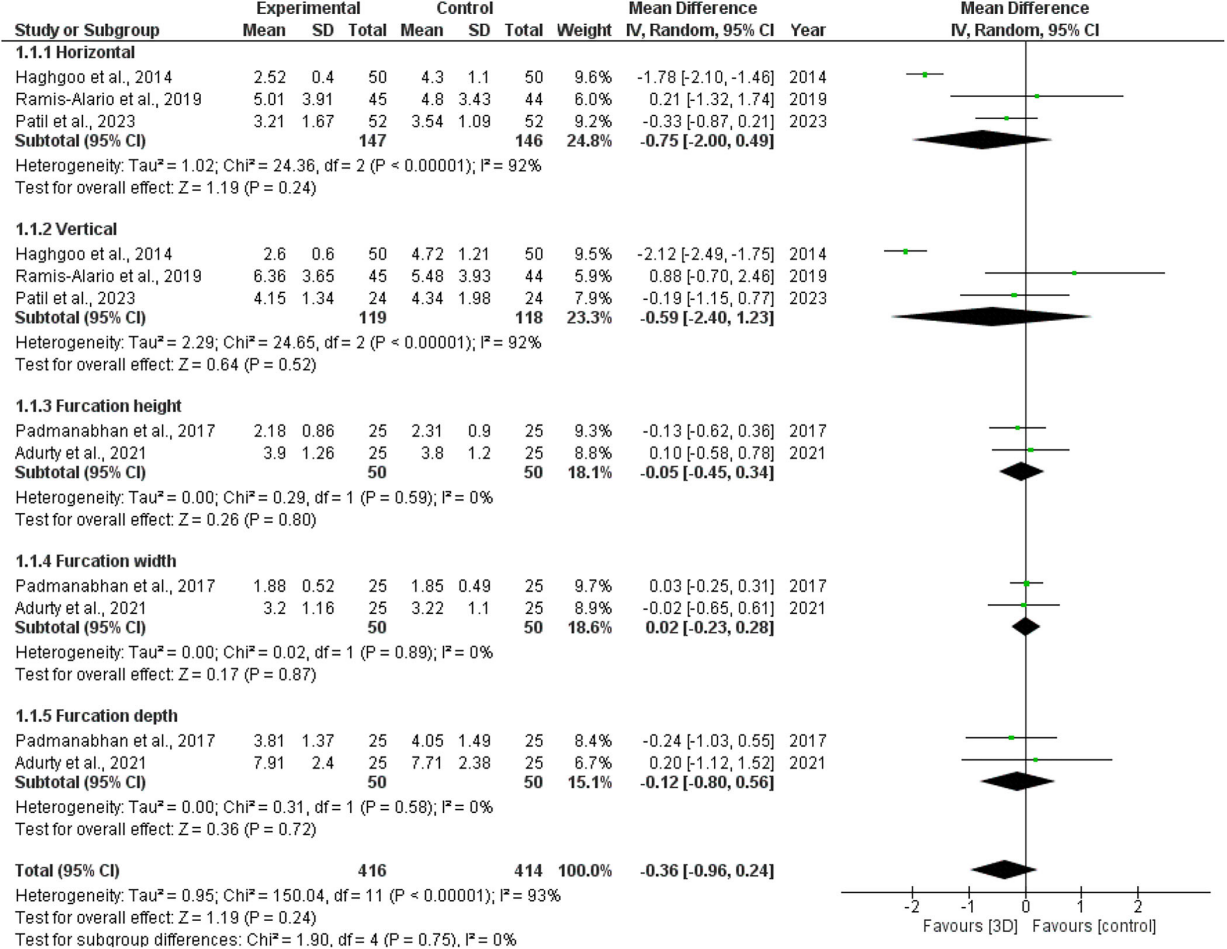
Forest plot for the effectiveness of 3D imaging over 2D.

Data variability led to a meta‐analysis of three studies across two subgroups (horizontal and vertical) and two studies across the subgroups of furcation height, width, and depth. The difference (*p* = 0.00001) was significant in horizontal measures for subgroups of 3D imaging versus other treatments (2D or intrasurgical), where the mean difference was −0.75 (95% CI: −2 to 0.49) and 92% heterogeneity. Vertical measures showed a significant difference (*p* = 0.00001), with a mean difference of −0.59 (95% CI: −2.40 to 1.23, *I* ^2^ = 92%) (Figure 4)

For the furcation height subgroup using 3D scanning with other techniques (2D or intra‐surgical), the difference was significant (*p* = 0.59) and was mean to be −0.05 (95% confidence interval [CI]: −0.45 to 0.34, *I* ^2^ = 0%). As far as furcation width was concerned, there was no significant difference (*p* = 0.89) with a mean difference of 0.02 (95% CI: −0.23 to 0.28, *I* ^2^ = 0%). The difference in furcation depth was nonsignificant (*p* = 0.58), with a mean of −0.12 (95% CI: −0.80 to 0.56, *I* ^2^ = 0%). The differences overall were large (*p* = 0.00001), averaged at −0.36 (95% CI: −0.96‐0.24, *I* ^2^ = 93%) (Figure 4).

### 3.7 Quality Assessment

**Figure 5:**
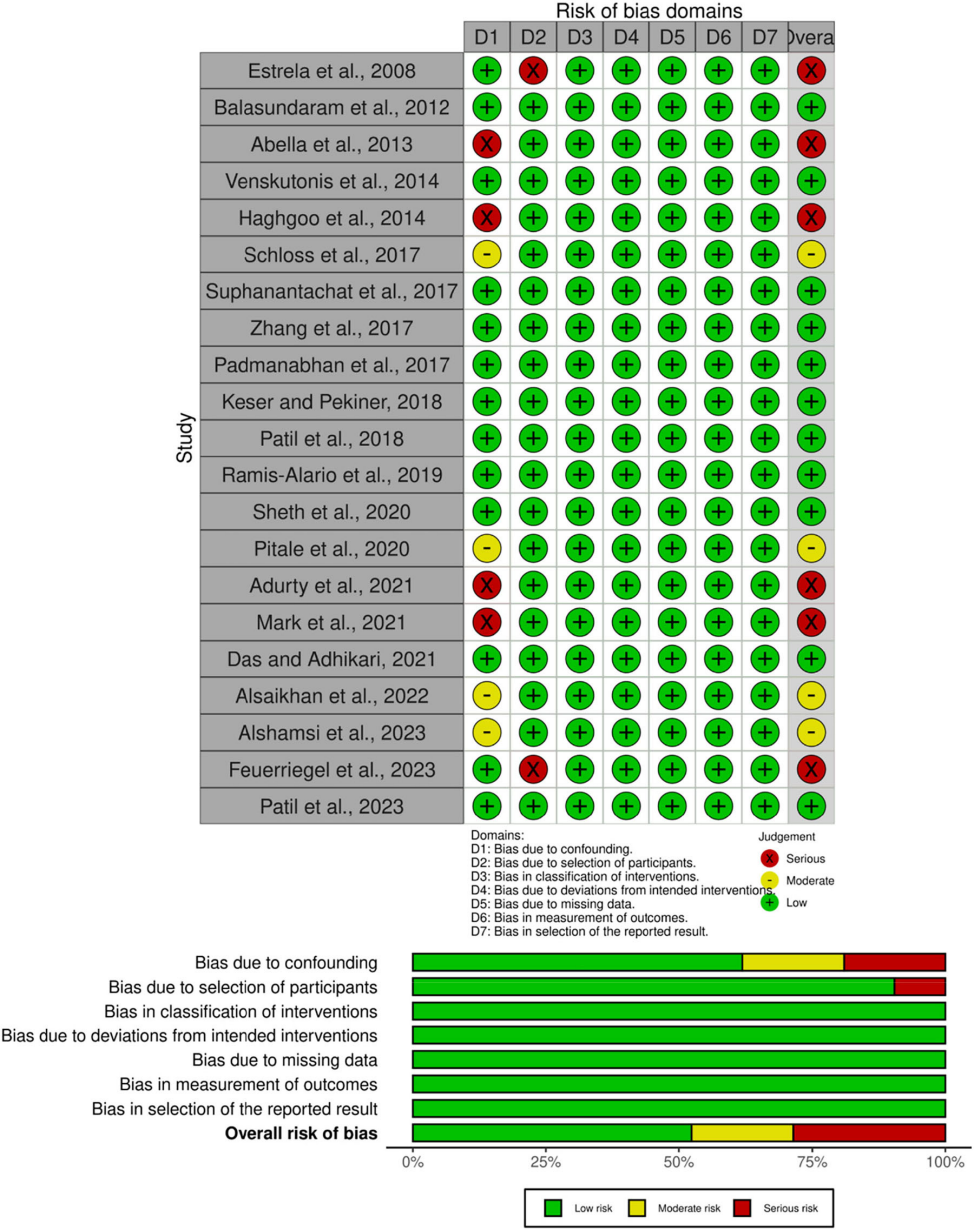
Quality assessment of non‐RCTs.

Using the ROBINS‐I quality assessment method, non‐RCTs were evaluated for biases. The majority of the studies were deemed to be of excellent quality, with a low risk of bias in areas such as confounding, participant choice, intervention classification, insufficient data, measurement of outcome, and outcomes reporting. However, four studies (Abella et al. 2013; Adurty et al. 2021; Moradi Haghgoo et al. 2014; Mark et al. 2021) had severe RoB in the domain of bias due to confounding, and two studies (Estrela et al. 2008; Feuerriegel et al. 2023) had a serious type of RoB in the domain of selection of participants (Figure 5). Furthermore, one RCT (Yusof et al. 2021) was found to be of good quality in all domains, with a low RoB.

### 3.8 Publication Bias

**Figure 6:**
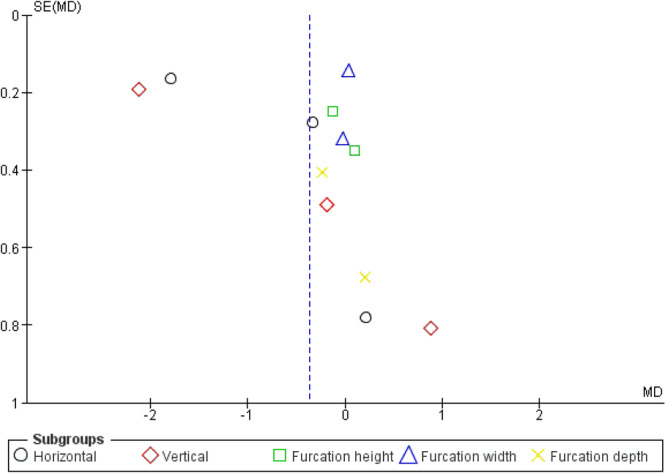
Funnel plot for publication bias.

The distribution of the studies appears somewhat asymmetrical, with most of the studies on one side of the line, which may suggest potential publication bias and heterogeneity among the included studies in the sub‐group analysis (Figure 6).

### 3.9 Meta‐Regression

Table 2 presents the results of a meta‐regression analysis examining the effect of different covariates on adherence rates. Among the covariates, age had a positive association with the heterogeneity (0.19, *p* = 0.008), Likewise, year of publication had also a positive association (0.16); however, statistically the impact was nonsignificant (*p* = 0.14). Study quality was significantly associated with negative effect (−1.51, *p* < 0.001), indicating that lower quality studies had an impact on the heterogeneity. Meanwhile, disease status (with periodontal disease or without periodontal disease) showed a positive nonsignificant association (1.82, *p* = 0.38) as described in Table 4.

**Table 4:** Meta‐regression outcomes‐random effect model.

Covariates	Point estimates	SE	95% CI	*Z* value	*p* value
Intercept	−8.45	2.81	−13.95 to −2.94	−3	0.003
Age	0.19	0.07	0.05–0.33	2.66	0.008
Intercept	−342.47	236.99	−06.97 to 122.03	−1.44	0.14
Year	0.16	0.11	−0.06 to 0.39	1.44	0.14
Intercept	−0.27	0.26	−0.78 to 0.24	−1.03	0.30
Study quality	−1.51	0.30	−2.11 to −0.90	−4.89	< 0.001
Intercept	−1.07	0.72	−2.49 to 0.34	−1.47	0.13
Disease status	1.82	1.46	−1.58 to 4.14	0.87	0.38

## 4 Discussion

Before starting any diagnosis, treatment planning, and prognosis of any periodontal disease, assessment of the bone condition has a significant value. In the field of periodontology, the most commonly used imaging modality is the 2D conventional and digital radiography. 2D methods are safe for patients because patients were not exposed to radiation during intra‐surgical measurements; however, they are unable to reveal the complex 3D architecture of periodontal diseases (Mohan et al. 2011). In addition, periodontal probes, which are considered a benchmark for the determination of periodontal defects in direct surgical measurements, are also used for the assessment of the periodontal health of patients. However, this method does not provide sufficient time to devise regenerative procedures (Chung et al. 2022). This systematic review and meta‐analysis sought to analyze the preciseness and accuracy of 3D imaging diagnostic techniques in evaluating periodontal disorders.

In the present study, the most identified 3D imaging tool is CBCT, which offers valuable advantages over other techniques (2D), including better accuracy and precision, and is justified as the best alternative diagnostic tool for the diagnosis of periodontal diseases for better treatment decisions (Komšić et al. 2019). Similarly, another systematic review revealed that CBCT is the best diagnostic imaging tool for the assessment of infrabony defects, alveolar bone crest height, furcation lesions, and periodontal ligament space (Choi et al. 2018). Other evidence also suggests that traditional radiographs have poor sensitivity compared to CBCT; however, these 2D radiographs have good diagnostic performance in terms of AUC_ROC_ and accuracy (Ramis‐Alario et al. 2021). Furthermore, CBCT had a significant (*p* < 0.05), strong, and positive association with intra‐oral parallel digital imaging with an intra‐class correlation coefficient of 0.93 (CBCT) and 0.78 (intra‐oral parallel digital imaging), which indicated that CBCT had superior accuracy (Abdinian et al. 2020). There are a few valuable reasons for its superiority over other techniques, such as volumetric data provision, which provides a 3D detailed structure of bones, allowing more localization, measurements, and assessment of periodontal diseases. It also provides images of more complex structures, which enable dentists to assess anatomical relationships and treatment plans with greater precision. In contrast, a systematic review was conducted to evaluate alveolar bone level based on the distance from the crest to the CEJ or gingival margin. The difference (mean) between the CBCT and ultrasound measurements ranged from 0.07 to 0.68 mm (1.6%–8.8%), which supports the use of ultrasound compared to CBCT (Nguyen et al. 2018). Bone loss (horizontal and vertical) CBCT is not a precise diagnostic tool for the assessment of developing bone problems (Oliveira et al. 2021). Although it provides 3D volumetric images, it also gives reconstructed axial, coronal, and sagittal multiplanar images without magnification. In addition, it enhances the 2D images without distortion (Acar 2014). These conflicting findings regarding the superiority of CBCT over 2D radiographs and ultrasound over CBCT can be attributed to differences in the diagnostics objectives, imaging principles and most importantly the study methodology and experience of the physicians in interpretation of the imaging outcomes. Furthermore, CBCT still has some limitations, the most vital of which is the higher dose of radiation compared to other 2D radiographs (Roberts et al. 2009). In CBCT, due to variability in the machine selection and the FOV size, the dose is also different: head (92.8 Sv, 206.2 Sv); 13 cm jaw scan (39.5 Sv, 133.9 Sv); 6 cm high‐resolution maxilla scan (18.5 Sv, 93.3 Sv); 6 cm mandible scan (47.2 Sv, 188.5 Sv); 6 cm mandible scan standard (23.9 Sv, 96.2 Sv) 6 cm standard scan of maxilla (9.7 Sv, 58.9 Sv)(Roberts et al. 2009). The radiation dose for CBCT is very critical as higher doses can pose health risks, when used routinely. Clinicians should weigh the benefits of CBCT against the potential risks, reserving its use for cases where conventional radiographs are not supposed to provide more precise diagnosis or treatment planning.

A meta‐analysis was conducted to assess the accuracy of the 3D imaging diagnostic modalities (CBCT and MRI). Unfavorably, because there was diversity in the data from the included studies, only a minimal number of studies were incorporated into the meta‐analysis. (Figure 4). Five subcategories existed, although only five investigations provided comparable results on horizontal and vertical disc/plane measures, as well as furcation height, width, and depth. There was a significant difference (*p* = 0.00001) between the horizontal and vertical subgroups, favoring the 3D imaging technique, whereas no significant difference was observed in the furcation height, width, and depth subgroups when comparing the 3D imaging technique to other methods (2D, intra‐surgical measurements). Our meta‐analysis findings cannot be compared with other meta‐analyses, and to our knowledge, no published meta‐analysis has compared 3D imaging techniques for the diagnosis of periodontal diseases with other 2D or intra‐surgical measurements. However, the significant difference in the case of horizontal and vertical disc/plane measurements may be due to their superior ability to provide more detailed spatial information, which ultimately allows for more accurate and precise measurements. However, nonsignificant differences in the furcation height, width, and depth subgroups may be due to several factors, such as complexities in the anatomy of furcation and imaging protocols used in the studies, which may introduce variability in the measurements. Furthermore, the expertise level of the investigators or radiologists also matters. Overall, there was a significant difference between both modalities, which indicated that the 3D modality had more accuracy and precision than the other modalities. However, certain disadvantages should also be considered when comparing CBCT and MRI, such as MRI is more expensive and limited in availability.

This study has several strengths as it provides a comprehensive evaluation of the most up‐to‐date literature on the utilization of 3D imaging for the assessment of periodontal diseases. The outcomes were presented qualitatively and quantitatively in the form of a meta‐analysis. Despite its strengths, our study has limitations, such as the lack of RCTs, which can affect the overall reliability of the conclusion of the present study, and the limited number of papers included in the meta‐analysis, which was due to the variation in data measurement and had a great impact on the outcomes of the meta‐analysis.

## 5 Conclusions

The current study highlights valuable information for the utilization of 3D imaging (CBCT and MRI) for the diagnostic assessment of periodontal diseases. Our qualitative and quantitative findings suggest that 3D imaging is more accurate and precise than other imaging (2D) or intra‐surgical measurement techniques. Additionally, according to the meta‐analysis, particularly in horizontal and vertical disc/plane measurements, it offers significant advantages, whereas, for furcation height, width, and depth, there was a nonsignificant difference, which underscores the need for further research. Notwithstanding this diversity, persistent trends support the use of 3D imaging in the evaluation of periodontal disorders. Nevertheless, owing to the negligible disparity in furcation height, width, and depth, the most significant advice is a careful integration of CBCT and digital intraoral radiography for improved periodontal bone evaluation. Future studies should concentrate on standardized procedures in comparison studies to yield uniform data for enhanced treatment planning of periodontal diseases. In addition, the cost‐effectiveness of CBCT vs 2D imaging modalities and the role of artificial intelligence in imaging studies should also be considered.

## Author Contributions

*Conceptualization*: Ravinder S. Saini and Sunil Kumar Vaddamanu. *Methodology*: Ravinder S. Saini and Sunil Kumar Vaddamanu. *Software*: Seyed Ali Mosaddad. *Validation*: Seyed Ali Mosaddad. *Formal analysis*: Ravinder S. Saini and Artak Heboyan. *Investigation*: Masroor Ahmed Kanji. *Resources*: Masroor Ahmed Kanji. *Data Curation*: Ravinder S. Saini and Artak Heboyan. *Writing – Original Draft*: Ravinder S. Saini and Artak Heboyan. *Writing – Review & Editing*: Ravinder S. Saini and Seyed Ali Mosaddad. *Supervision*: Artak Heboyan. *Project administration:* Artak Heboyan. *Funding acquisition*: Ravinder S. Saini. All authors have read and approved the published version of the manuscript.

## Ethics Statement

The authors have nothing to report.

## Consent

The authors have nothing to report.

## Conflicts of Interest

The authors declare no conflicts of interest.

## Supporting information

Supp_Table.

## Data Availability

The data that support the findings of this study are available from the corresponding author upon reasonable request.
