# Supplementary material for: Comparative Analysis of 3D Imaging in Periodontal Disease Assessment: A Systematic Review and Meta‐Analysis
Source: Clin Exp Dent Res. 2025 Jul 13;11(4):e70169. doi: 10.1002/cre2.70169 (PMC12256084; doi:10.1002/cre2.70169)
Supplement: Supplementary file 1 — Supp_Table. [file CRE2-11-e70169-s001.docx]

**Supplementary table 1.** Literature searched from different databases.

| Databases | Search terms |
| --- | --- |
| PubMed | ("Three dimensional imaging"[Title/Abstract] OR "3D imaging"[Title/Abstract] OR "Cone beam computed tomography"[MeSH Terms] OR "Cone beam CT"[Title/Abstract] OR "CBCT"[Title/Abstract] OR "Magnetic resonance imaging"[MeSH Terms] OR "MRI"[Title/Abstract] OR "Dental computed tomography"[Title/Abstract] OR "Digital volume tomography"[Title/Abstract]) AND ("Periodontal disease"[Title/Abstract] OR "Periodontitis"[Title/Abstract] OR "Periapical disease"[Title/Abstract] OR "Periapical lesion"[Title/Abstract] OR "Apical periodontitis"[Title/Abstract] OR "Alveolar bone loss"[Title/Abstract] OR "Horizontal bone loss"[Title/Abstract] OR "Vertical bone loss"[Title/Abstract] OR "Furcation defects"[MeSH Terms] OR "Furcation involvement"[Title/Abstract] OR "Intrabony defect"[Title/Abstract] OR "Gingivitis"[MeSH Terms]) |
| ScienceDirect | ("Three-dimensional imaging" OR "3D imaging" OR "Cone beam computed tomography" OR "Magnetic resonance imaging") AND ("Periodontal disease" OR "Periodontitis" OR "Alveolar bone loss" OR "Furcation defects" OR "Gingivitis") |
| Google Scholar | (“Three-dimensional imaging”\|“3D imaging ”\|“Cone beam computed tomography”\|“Cone beam CT”\|“CBCT”\|“Magnetic resonance imaging”\|“MRI”) (“Periodontal disease”\|“Periodontitis”\|“Periapical disease”\|“Alveolar bone loss”\|“Furcation defects”\|“Gingivitis”) |
| Scopus | ("Three dimensional imaging" OR "3D imaging" OR "Cone beam computed tomography" OR "Cone beam CT" OR "CBCT" OR "Magnetic resonance imaging" OR "MRI" OR "Dental computed tomography" OR "Digital volume tomography") AND ("Periodontal disease" OR "Periodontitis" OR "Periapical disease" OR "Periapical lesion" OR "Apical periodontitis" OR "Alveolar bone loss" OR "Horizontal bone loss" OR "Vertical bone loss" OR "Furcation defects" OR "Furcation involvement" OR "Intrabony defect" OR "Gingivitis") |
| Web of Sciences | ("Three dimensional imaging" OR "3D imaging" OR "Cone beam computed tomography" OR "Cone beam CT" OR "CBCT" OR "Magnetic resonance imaging" OR "MRI" OR "Dental computed tomography" OR "Digital volume tomography") AND ("Periodontal disease" OR "Periodontitis" OR "Periapical disease" OR "Periapical lesion" OR "Apical periodontitis" OR "Alveolar bone loss" OR "Horizontal bone loss" OR "Vertical bone loss" OR "Furcation defects" OR "Furcation involvement" OR "Intrabony defect" OR "Gingivitis") |
